# Supplementary material for: Evaluation of Mono- and Bi-Functional GLOBE-Based Vectors for Therapy of β-Thalassemia by HBBAS3 Gene Addition and Mutation-Specific RNA Interference
Source: Cells. 2023 Dec 15;12(24):2848. doi: 10.3390/cells12242848 (PMC10741507; doi:10.3390/cells12242848)
Supplement: Supplementary file 1 [file cells-12-02848-s001.zip › Koniali_et_al_LV_IVSI-110_sup_20231214.pdf]

cells

Supplementary Materials

Evaluation of Mono- and Bi-Functional GLOBE-Based Vectors for the Therapy of β-Thalassemia via *HBB*^{AS3} Gene Addition and Mutation-Specific RNA Interference

Lola Koniali [1], Christina Flouri [2,†], Markela I. Kostopoulou [1], Nikoletta Y. Papaioannou [1], Panayiota L. Papasavva [1], Basma Naiisseh [1], Coralea Stephanou [1], Anthi Demetriadou [1], Maria Sitarou [3], Soteroula Christou [4], Michael N. Antoniou [2], Marina Kleanthous [1], Petros Patsali [1,*] and Carsten W. Lederer [1,*]

[1] Department of Molecular Genetics Thalassaemia, The Cyprus Institute of Neurology & Genetics, 6 Iroon Avenue, 2371 Nicosia, Cyprus; lkoniali@hotmail.com (L.K.); markella.costopoulou@hotmail.com (M.I.K.); nikolettap@cing.ac.cy (N.Y.P.); panayiotap@cing.ac.cy (P.L.P.); basman@cing.ac.cy (B.N.); coraleas@cing.ac.cy (C.S.); anthide@cing.ac.cy (A.D.); marinakl@cing.ac.cy (M.K.)

[2] Gene Expression and Therapy Group, Department of Medical and Molecular Genetics, King's College London, Guy's Hospital, London SE1 9RT, UK; christina.flouri@gmail.com (C.F.); michael.antoniou@kcl.ac.uk (M.N.A.)

[3] Thalassemia Clinic Larnaca, Larnaca General Hospital, 6301 Larnaca, Cyprus; msitarou@yahoo.gr

[4] Thalassemia Clinic Nicosia, Archbishop Makarios III Hospital, 1474 Nicosia, Cyprus; snchrthalcl@cytanet.com.cy

* Correspondence: petrospa@cing.ac.cy (P.P.); lederer@cing.ac.cy (C.W.L.); Tel.: +357-22-392652 (C.W.L.); Tel.: +357-22-392764 (P.P.)

† Current address: Medicines Research Center, GSK, Stevenage SG1 2NY, UK.

Citation: Koniali, L.; Flouri, C.; Kostopoulou, M.I.; Papaioannou, N.Y.; Papasavva, P.L.; Naiisseh, B.; Stephanou, C.; Demetriadou, A.; Sitarou, M.; Christou, S.; et al. Evaluation of Mono- and Bi-Functional GLOBE-Based Vectors for the Therapy of β-Thalassemia via *HBB*^{AS3} Gene Addition and Mutation-Specific RNA Interference. *Cells* **2023**, *12*, 2848. https://doi.org/10.3390/cells12242848

Academic Editors: Albert Rizvanov and Ayşegül Doğan

Received: 15 October 2023
Revised: 10 December 2023
Accepted: 11 December 2023
Published: 15 December 2023

Copyright: © 2023 by the authors. Licensee MDPI, Basel, Switzerland. This article is an open access article distributed under the terms and conditions of the Creative Commons Attribution (CC BY) license (https://creativecommons.org/licenses/by/4.0/).

1. Supplementary Figures

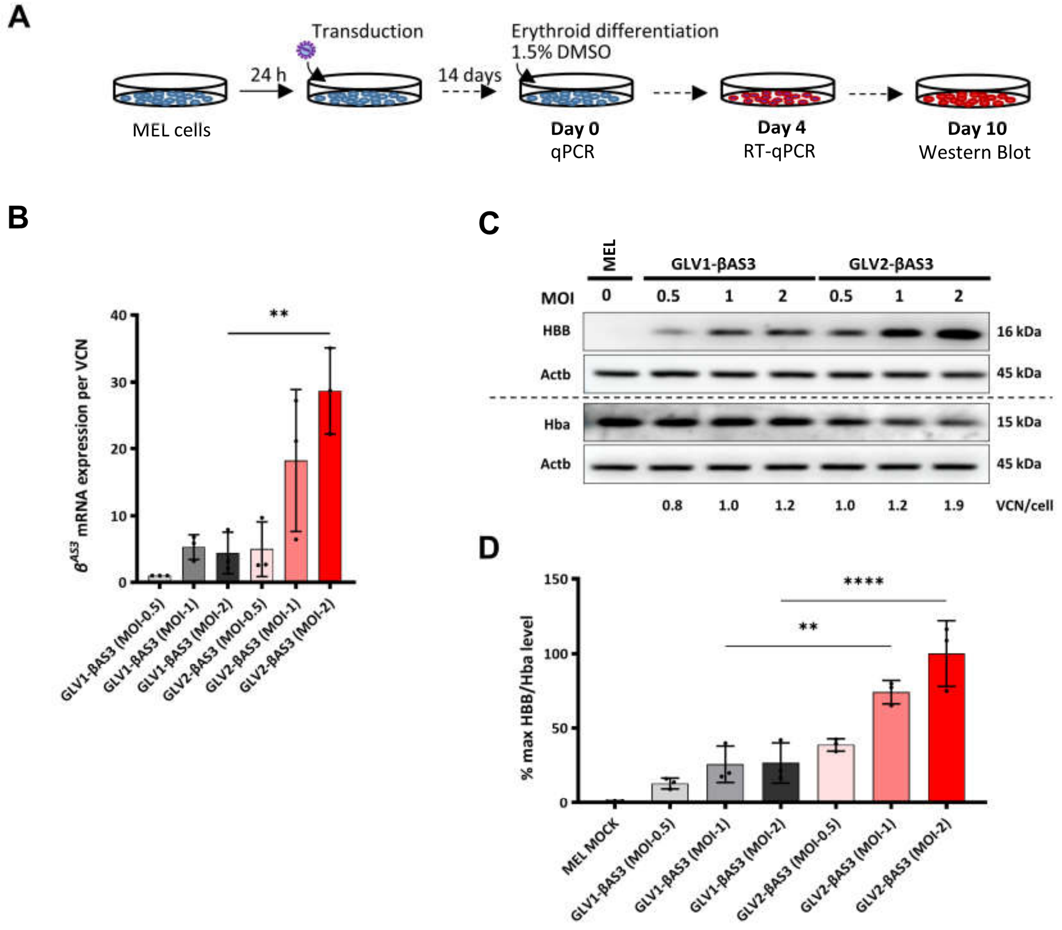

Figure S1. Functional analysis of the GLOBE-based HBB^{AS3} transgene-expressing lentiviral vectors in MEL cells. (**A**) MEL cell transduction with GLV1-βAS3 and GLV2-βAS3 at increasing MOI (0.5, 1, 2) and erythroid differentiation for 10 days to assess vector-derived β^{AS3}-transgene expression levels. (**B**) Relative HBB^{AS3} (β^{AS3}) mRNA expression measured by RT-qPCR on day 4 of erythroid differentiation (n=3). (**C**) Immunoblots of HBB, Hba protein and β-actin–used as loading control in terminally differentiated (day 10) mock- and LV-transduced MEL cell pools (n=3) , based on equivalent detection of HBB and HBB^{AS3} by anti-HBB antibody. The dashed line indicates detection on separate membranes. (**D**) Percentage of differentiation-normalized HBB chain levels relative to the highest value for each experiment, as determined via densitometry analysis using Image J. Messenger RNA and protein levels were normalized to endogenous Hba expression and corrected for VCN. Statistical significance was calculated using one-way ANOVA, * $p<0.05$ ** $p < 0.01$ *** $p<0.001$ and ****<0.0001.

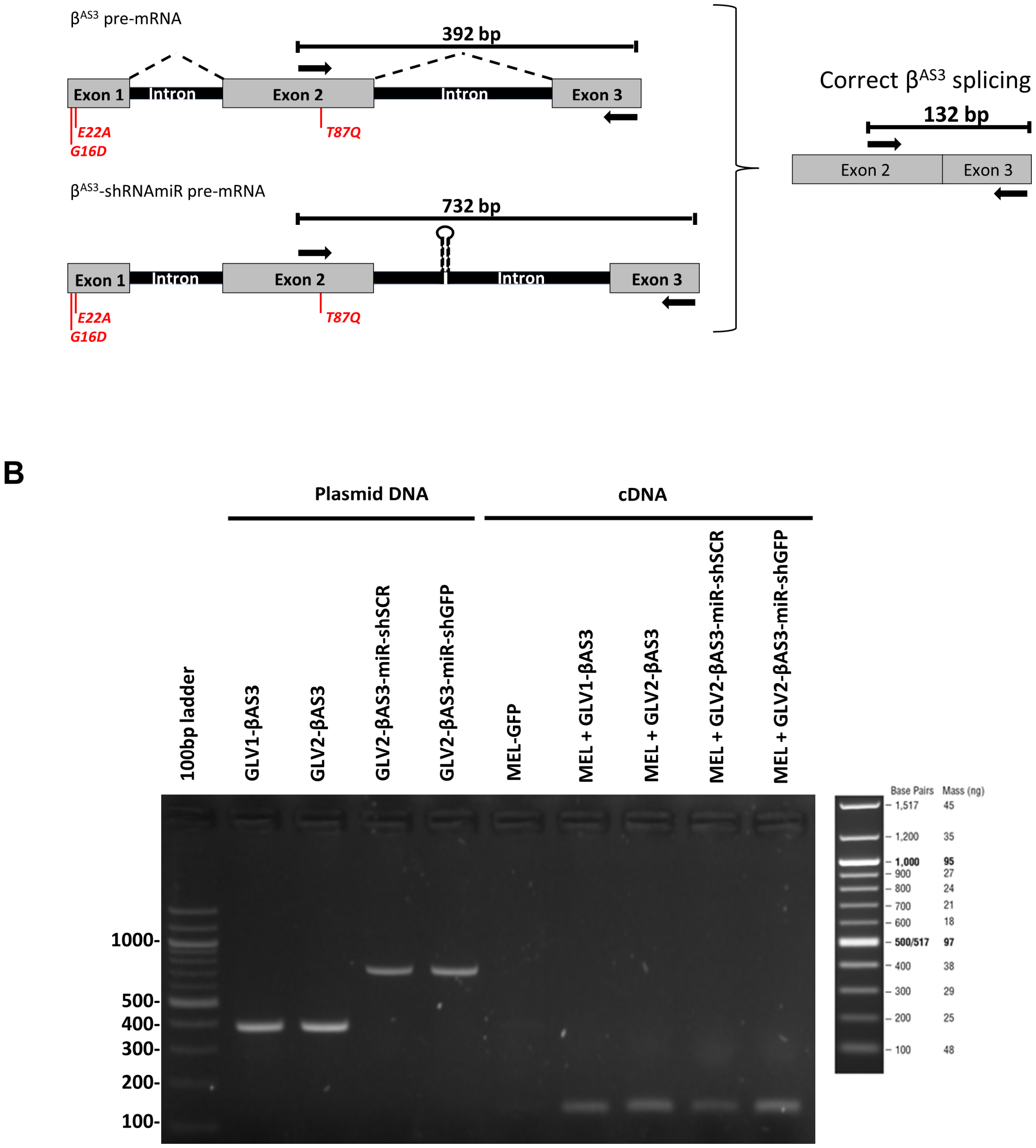

Figure S2. Splicing of the anti-sickling human *HBB*^{AS3} transgene bearing the miR30-shRNA expression cassette. (**A**) Schematic of *HBB*^{AS3} (β^{AS3}) transgene (exon 1 and exon 2 are represented as grey boxes, and intron 1 and 2 as black lines) bearing the three anti-sickling mutations (indicated in red) with the intron-encoded miR30-expression cassette (βAS3-miR30-shRNA pre-mRNA). Binding regions of PCR primers specific for β^{AS3} fragment detection are shown with black arrows. (**B**) PCR products were separated by gel electrophoresis on a 2% agarose gel.

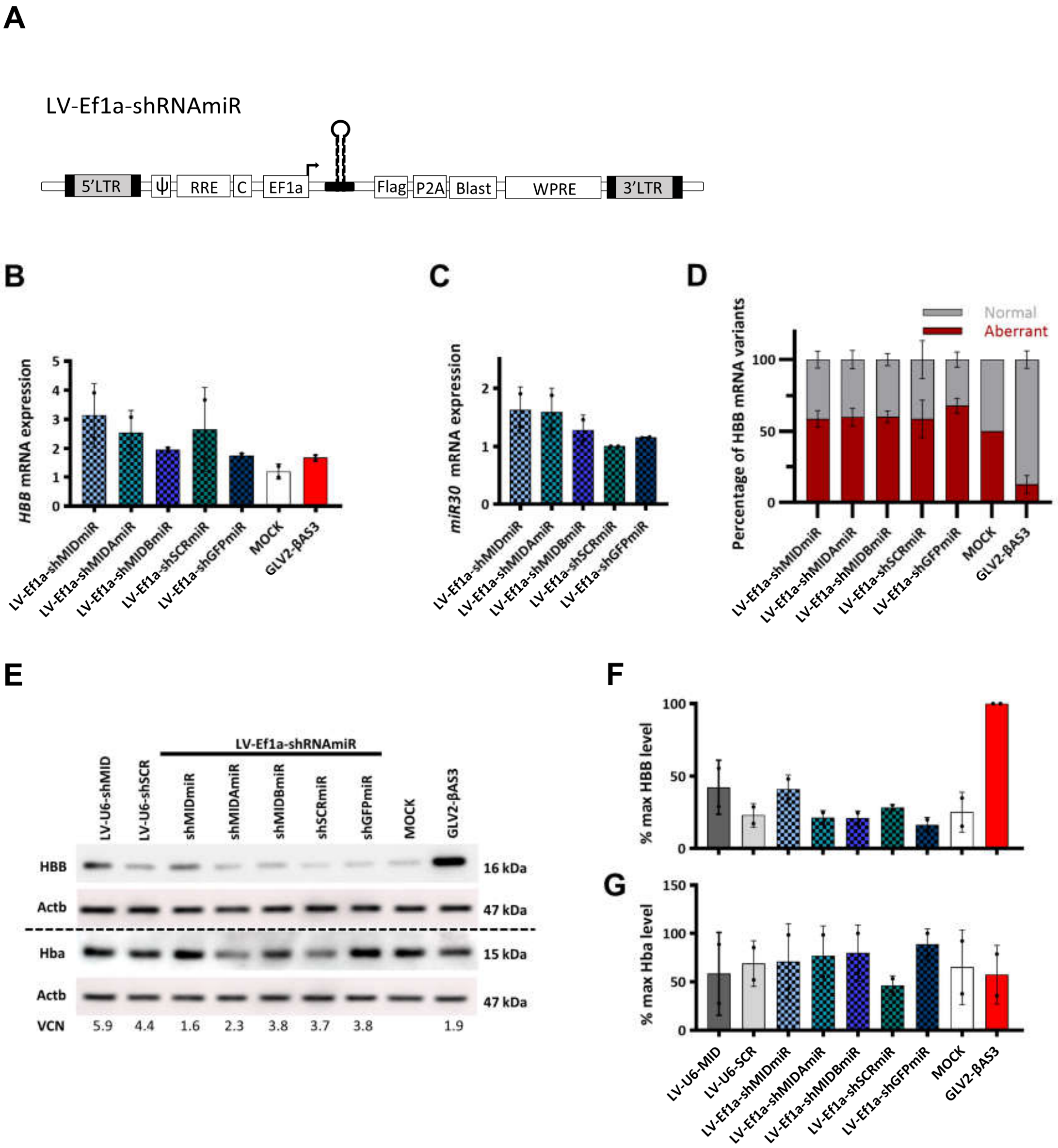

Figure S3. Design and functional evaluation of shRNAmiR guide strand variants targeting the aberrant $HBB^{IVSI-110(G>A)}$ mRNA using the *Ef1a*-promoter-driven LV (LV-Ef1a-shRNAmiR). (**A**) Schematic representation of lentiCas9-Blast (Addgene: 52962) modified to express the miR30-based shRNA expression cassette in place of Cas9. ψ, Psi packaging signal; RRE, Rev response element; C, central polypurine tract; EF1a, elongation factor 1a short promoter; Flag, Flag octapeptide tag; P2A, 2A self-cleaving peptide; WPRE, Woodchuck Hepatitis Virus post-transcriptional regulatory element; Blast, blasticidin selection marker; 5′/3′LTR, 5′ and 3′ LV long terminal repeats. (**B**) Relative *HBB* mRNA expression and **(C)** vector-derived miR30 mRNA expression measured by RT-qPCR on day 4 of erythroid differentiation of MEL-HBB^{IVS} cells. Target mRNA levels were normalized against *Hba* mRNA and expressed per vector copy. Data represent mean±SD of three independent experiments from a single LV preparation. (**D**) Percentage contribution of aberrant and normal *HBB* mRNA in bulk MEL-HBB^{IVS} cells (n=3). (**E**) Representative images of immunoblots for HBB, Hba protein levels and β-actin (used as loading control) in transduced MEL-HBB^{IVS} cells on day 10 of erythroid differentiation, based on equivalent detection of HBB and HBB^{AS3} by anti-HBB antibody. The dashed line indicates detection on separate membranes. **(F)** Percentage of differentiation-normalized HBB levels (normalized to VCN) and (**G**) β-actin-normalized Hba levels relative to the highest value for each experiment as determined by densitometry analysis of immunoblots from two independent transduction experiments.

2. Supplementary Tables

Table S1. Oligonucleotides used for the generation of miR30^shRNA-based vectors

Oligonucleotides	Sequence (5′-3′)[1]
miR30^shMID	cgtctcaagcgagtctattttcccacccttaggtagtgaagccacagatgtacctaagggtgggaaaatagacctgcctgagacg
miR30^shMIDA	cgtctcaagcgagcgcgtctattttcccacccttagtgaagccacagatgtaagggtgggaaaatagacgcgcctgcctgagacg
miR30^shMIDB	cgtctcaagcgagcgcattttcccacccttaggtagtgaagccacagatgtacctaagggtgggaaaatgcgcctgcctgagacg
miR30^shSCR	cgtctcaagcgacctaaggttaagtcgccctcgctctagtgaagccacagatgtagagcgagggcgacttaaccttaggctgcctgagacg
miR30^shGFP	cgtctcaagcgagcacaagctggagtacaactatagtgaagccacagatgtatagttgtactccagcttgtgcctgcctgagacg
β-Term sequence	gcatagtgttaccatcaaccaccttaacttcatttttcttattcaatacctaggtaggtagatgctagattctggaaataaaatatgagtctcaagtgg<br>tccttgtcctctctcccagtcaaattctgaatctagttggcaagattctgaaatcaaggcatataatcagtaataagtgatgatagaagggtatatag<br>aagaattttattatatgagagggtgaaaccctcaaaatgaaatgaaatcagacccttgtcttacaccataaacaaaaataaatttgaatgggttaa<br>agaattaaactaagacctaaaaccataaaaatttttaaagaaatcaaaagaagaaaattctaatattcatgttgcagccgtttttgaatttgatatg<br>agaagcaaaggcaacaaaaggaaaaataaagaagtgaggctacatcaaactaaaaaatttccacacaaaaaagaaaacaatgaacaaatga<br>aaggtgaaccatgaaatggcatatttgcaaaccaaatatttcttaaatattttggttaatatccaaaatatataagaaacacagatgattcaataaca<br>aacaaaaaattaaaaataggaaaataaaaaaattaaaaagaagaaaatcctgccatttatgcgagaattgatgaacctggaggatgtaaaact<br>aagaaaaataagcctgacacaaaaagacaaatactacacaaccttgctcatatgtgaaacataaaaaagtcactctcatggaaacagacagtag<br>aggtatggtttccaggggttgggggtgggagaatcaggaaac
Construct 1: miR30shRNA expression cassette with inverted *Bsm*BI sites	atcgataccgtgtaaatacacttgcaaaggaggatgtttttagtagcaatttgtactgatggtatggggccaagagatatatcttagagggaggg<br>ctgagggtttgaagtccaactcctaagccagtgccagaagagccaaggacaggtacggctgtcatcacttagacctcaccctgtggagccacac<br>cctagggttggccaatctactcccaggagcagggagggcaggagccagggctgggcataaaagtcagggcagagccatctattgcttacattt<br>gcttctgacacaactgtgttcactagcaacctcaaacagacaccaccggttgtttgaatgaggcttcagtactttacagaatcgttgcctgcacatct<br>tggaaacacttgctgggattacttcttcaggttaacccaacagaaggctaaagaaggtatattgctgttgacagtgagcgagagacggaagccc<br>agacgtctcatgcctactgcctcggacttcaaggggctactttaggagcaattatcttgtttactaaaactgaataccttgctatctctttgatacattt<br>ttacaaagctgaattaaaatggtataaattaaatcacttttagcgctgctcgctttcttgctgtccaatttctattaaaggttcctttgttccctaagtcca<br>actactaaactggggggatattatgaagggccttgagcatctggattctgcctaataaaaaacatttattttcattgcaatgatgtatttaaat
Construct 2: β^AS3-globin transgene with AgeI and AfeI restriction sites in IVS2 break point	atcgataccgtgtaaatacacttgcaaaggaggatgtttttagtagcaatttgtactgatggtatggggccaagagatatatcttagagggaggg<br>ctgagggtttgaagtccaactcctaagccagtgccagaagagccaaggacaggtacggctgtcatcacttagacctcaccctgtggagccacac<br>cctagggttggccaatctactcccaggagcagggagggcaggagccagggctgggcataaaagtcagggcagagccatctattgcttacattt<br>gcttctgacacaactgtgttcactagcaacctcaaacagacaccatggtgcacctgactcctgaggagaagtctgccgttactgccctgtgggac<br>aaggtgaacgtggatgccgttggtggtgaggccctgggcaggttggtatcaaggttacaagacaggtttaaggagaccaatagaaactgggc<br>atgtggagacagagaagactcttgggtttctgataggcactgactctctctgcctattggtctattttcccacccttaggctgctggtggtctaccctt<br>ggacccagaggttctttgagtcctttggggatctgtccactcctgatgctgttatgggcaaccctaaggtgaaggctcatggcaagaaagtgctc<br>ggtgcctttagtgatggcctggctcacctggacaacctcaagggcacctttgcccagctgagtgagctgcactgtgacaagctgcacgtggatcc<br>tgagaacttcagggtgagtctatgggacccttgatgttttctttccccttcttttctatggttaagttcatgtcataggaaggggagaagtaacaggg<br>taaccggttctaggccaccagcgctttctgcatataaattgtaactgatgtaagaggtttcatattgctaatagcagctacaatccagctaccattct<br>gcttttattttatggttgggataaggctggattattctgagtccaagctaggcccttttgctaatcatgttcatacctcttatcttcctcccacagctcctg<br>ggcaacgtgctggtctgtgtgctggcccatcactttggcaaagaattcaccccaccagtgcaggctgcctatcagaaagtggtggctggtgtgg<br>ctaatgccctggcccacaagtatcactaagctcgctttcttgctgtccaatttctattaaaggttcctttgttccctaagtccaactactaaactggggg<br>atattatgaagggccttgagcatctggattctgcctaataaaaaacatttattttcattgcaatgatgtatttaaat

[1]Underlined sequences indicate restriction sites used for cloning.

Table S2. Primers and Probes used for PCR-based assays

Assay	Primer	Sequence (5′-3′)
Vector copy number	LV_Fw	TCTCGACGCAGGACTCG
	LV_Rv	TACTGACGCTCTCGCACC
	LV Probe	Yakima-Yellow-ATCTCTCTCCTTCTAGCCTC- ZNA-4-BHQ-1
	PCB2_Fw	TTGTGTCTCCAGTCTGCTTG
	PCB2_Rv	AGGTGGTGGTGGTGGTA
	PCB2 Probe	FAM-CCCTCTCCTGGCTCTAAATGTTGTGT- BHQ-1
Aberrant and normal β-globin mRNA expression	hHBB_EX1_Fw	GGGCAAGGTGAACGTG
	hHBB_EX2_Rv	GGACAGATCCCCAAAGGAC
	IVS1-110 MGB Probe	VIC-TGGGCAGTCTATTT-MGB-NFQ
	wtHBB ZNA Probe	6-FAM- TGG G(PDC)A GG(PDC) TG(PDC) TG-ZNA-3-BHQ-1
Human α-globin mRNA expression	HAlpha_Fw	GGACCCGGTCAACTTCAA
	HAlpha_Rv	CGGTATTTGGAGGTCAGCAC
Murine α-globin mRNA expression	hAlpha_Fw	GTCACGGCAAGAAGGTCGC
	hAlpha_Rv	GGGGTGAAATCGGCAGGGT
Human β-globin mRNA ex-	hHBB_EX2_Fw	GGCAAGAAAGTGCTCGG
	hHBB_EX2.3_Rv	GTGCAGCTCACTCAGTG
Vector derived βAS3-globin mRNA expression	LV_βAS3_G16D_Fw	AAGGTGAACGTGGATGCCG
	LV_βAS3_Rv	GCACTTTCTTGCCATGAGCC
Vector derived miR30 mRNA expression	LV_miR30_Fw	AATCGTTGCCTGCACATCTTG
	LV_miR30_Rv	CCTTCTTTAGCCTTCTGTTGGG
Vector derived eGFP mRNA expression	eGFP_Fw	GGCAAGCTGACCCTGAAGTT
	eGFP_Rv	AGATGGTGCGCTCCTGGA
βAS3-globin mRNA splicing	LV_βAS3_T87Q_Fw	CAAGGGCACCTTTGCCCAG
	LV_βAS3_EX3_Rv	GGTGGGGTGAATTCTTTGCC

Disclaimer/Publisher's Note: The statements, opinions and data contained in all publications are solely those of the individual author(s) and contributor(s) and not of MDPI and/or the editor(s). MDPI and/or the editor(s) disclaim responsibility for any injury to people or property resulting from any ideas, methods, instructions or products referred to in the content.
